# A Web-Based, Mobile-Responsive Application to Screen Health Care Workers for COVID-19 Symptoms: Rapid Design, Deployment, and Usage

**DOI:** 10.2196/19533

**Published:** 2020-10-08

**Authors:** Haipeng Zhang, Dimitar Dimitrov, Lynn Simpson, Nina Plaks, Balaji Singh, Stephen Penney, Jo Charles, Rosemary Sheehan, Steven Flammini, Shawn Murphy, Adam Landman

**Affiliations:** 1 Digital Innovation Hub Brigham and Women's Hospital Boston, MA United States; 2 Department of Psychosocial Oncology and Palliative Care Dana-Farber Cancer Institute Boston, MA United States; 3 Department of Medicine Harvard Medical School Boston, MA United States; 4 Research Information Science and Computing Partners HealthCare Boston, MA United States; 5 Partners Information Systems Partners HealthCare Somerville, MA United States; 6 Partners Enterprise Data and Digital Health Partners HealthCare Boston, MA United States; 7 Partners Human Resources Partners HealthCare Somerville, MA United States; 8 Department of Neurology Massachusetts General Hospital Harvard Medical School Boston, MA United States; 9 Department of Emergency Medicine Brigham and Women’s Hospital Harvard Medical School Boston, MA United States

**Keywords:** public health, clinical informatics, digital health, coronavirus, COVID-19, SARS-CoV-2, 2019-nCov, app, eHealth

## Abstract

**Background:**

As of July 17, 2020, the COVID-19 pandemic has affected over 14 million people worldwide, with over 3.68 million cases in the United States. As the number of COVID-19 cases increased in Massachusetts, the Massachusetts Department of Public Health mandated that all health care workers be screened for symptoms daily prior to entering any hospital or health care facility. We rapidly created a digital COVID-19 symptom screening tool to enable this screening for a large, academic, integrated health care delivery system, Partners HealthCare, in Boston, Massachusetts.

**Objective:**

The aim of this study is to describe the design and development of the COVID Pass COVID-19 symptom screening application and report aggregate usage data from the first three months of its use across the organization.

**Methods:**

Using agile principles, we designed, tested, and implemented a solution over the span of one week using progressively customized development approaches as the requirements and use case become more solidified. We developed the minimum viable product (MVP) of a mobile-responsive, web-based, self-service application using research electronic data capture (REDCap). For employees without access to a computer or mobile device to use the self-service application, we established a manual process where in-person, socially distanced screeners asked employees entering the site if they have symptoms and then manually recorded the responses in an Office 365 Form. A custom .NET Framework application solution was developed as COVID Pass was scaled. We collected log data from the .NET application, REDCap, and Microsoft Office 365 from the first three months of enterprise deployment (March 30 to June 30, 2020). Aggregate descriptive statistics, including overall employee attestations by day and site, employee attestations by application method (COVID Pass automatic screening vs manual screening), employee attestations by time of day, and percentage of employees reporting COVID-19 symptoms, were obtained.

**Results:**

We rapidly created the MVP and gradually deployed it across the hospitals in our organization. By the end of the first week, the screening application was being used by over 25,000 employees each weekday. After three months, 2,169,406 attestations were recorded with COVID Pass. Over this period, 1865/160,159 employees (1.2%) reported positive symptoms. 1,976,379 of the 2,169,406 attestations (91.1%) were generated from the self-service screening application. The remainder were generated either from manual attestation processes (174,865/2,169,406, 8.1%) or COVID Pass kiosks (25,133/2,169,406, 1.2%). Hospital staff continued to work 24 hours per day, with staff attestations peaking around shift changes between 7 and 8 AM, 2 and 3 PM, 4 and 6 PM, and 11 PM and midnight.

**Conclusions:**

Using rapid, agile development, we quickly created and deployed a dedicated employee attestation application that gained widespread adoption and use within our health system. Further, we identified 1865 symptomatic employees who otherwise may have come to work, potentially putting others at risk. We share the story of our implementation, lessons learned, and source code (via GitHub) for other institutions who may want to implement similar solutions.

## Introduction

To date, over 14 million cases of COVID-19 have been confirmed worldwide, with over 3.68 million cases in the United States [1]. This number continues to grow, and the United States has become the epicenter of COVID-19 [2]. By April 5, 2020, in the Commonwealth of Massachusetts, there were already 12,500 confirmed cases of COVID-19 and 231 deaths [3]. With exponentially increasing numbers of COVID-19 cases, the need for digital technology to address issues arising from pandemics such as COVID-19 has grown considerably [4].

To limit the spread and “flatten the curve,” on March 16, 2020, the Massachusetts Department of Public Health (MDPH) and the Commissioner of Public Health issued an order that Massachusetts hospitals must screen all visitors, including employees, for symptoms of a respiratory infection (fever, cough, shortness of breath, or sore throat) and that individuals with any symptoms should not be permitted to visit the hospitals [5]. Shortly after, our institution enacted a policy that all employees working in a patient care facility must wear a face mask while working as another measure to limit the spread of COVID-19 within the health care workforce [6].

We created a digital symptom screening and attestation tool, called COVID Pass, that provides daily facility passes and face mask passes for employees as its output. We used a prototype-driven innovation model combined with a transition to a more traditional custom development team as the application requirements matured, which allowed our group to rapidly deploy and refine the solution as it was released and approached scale. In the first 2 weeks, COVID Pass transitioned from a paper proposal to an enterprise-supported solution used by over 25,000 employees daily. After 3 months, over 2 million attestations were performed using the application.

In this paper, we describe the design, development, and use of the COVID Pass application, and we make the code available for other institutions seeking to implement a similar solution.

## Methods

This study was conducted at Partners HealthCare, a not-for-profit, academic, integrated health care delivery system in Boston, Massachusetts. Partners include Brigham and Women’s Hospital, Massachusetts General Hospital, community and specialty hospitals, a physician network, community health centers, home care, a health insurance plan, and other health-related services. The largest private employer in Massachusetts, Partners HealthCare has approximately 74,000 employees, including physicians, nurses, scientists, and caregivers.

Complying with the MDPH requirement for symptom screening in such a large, widely geographically distributed organization was expected to be difficult. The Partners HealthCare Chief Human Resources Officer recognized an opportunity to link the distribution of masks (something that employees wanted) with completion of the symptom screening (something that might be more difficult to have all employees complete). The Human Resources team and Occupational Health Services provided the initial requirements, including an application that would enable employees who must work on-site at a facility that provides direct patient care to be able to self-screen for symptoms of COVID-19 infection prior to being allowed to enter the facility. This application needed to be mobile-responsive, provide guidance to the employee about next steps if they indicated symptoms were present, create a pass that would be “glanceable” to entrance way screening staff, and be capable of exporting user logs on at least a daily basis. A manual pathway where on-site screeners would ask employees about symptoms and record them was also created to address the needs of employees who were unable to use the electronic self-screening tool.

Using agile principles, we designed, tested, and implemented a solution over the span of a week using progressively customized development approaches as the requirements and use case become more solidified. Based on the requirements, we developed the minimum viable product (MVP) of the self-service application using research electronic data capture (REDCap) due to the speed at which we could develop a functional prototype using the prebuilt data export systems of this solution. REDCap is a secure, web-based software platform that is designed to support data capture for research studies, providing an intuitive interface for validated data capture; audit trails for tracking data manipulation and export procedures; automated export procedures for seamless data downloads to common statistical packages; and procedures for data integration and interoperability with external sources [7,8].

**Figure 1:**
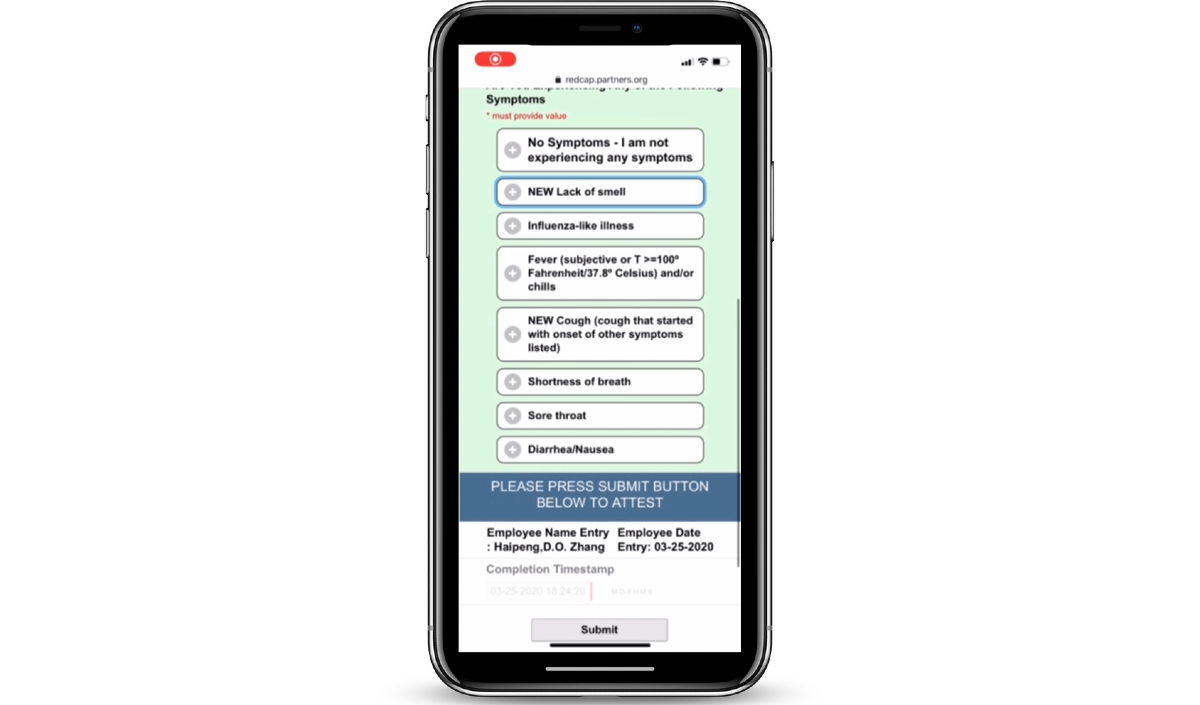
Employee symptom reporting screen of the COVID Pass application.

**Figure 2:**
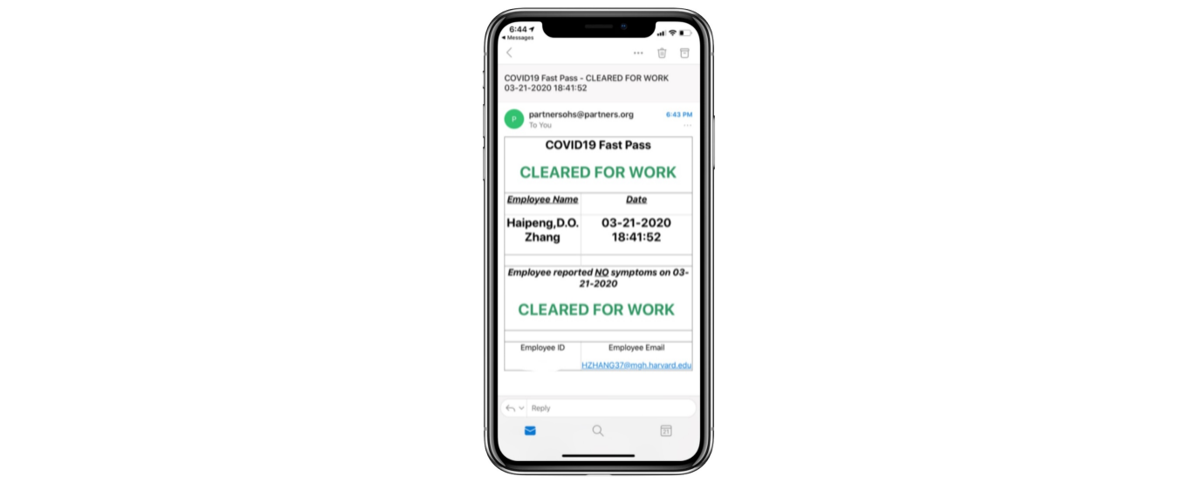
“Cleared for Work” screen of the COVID Pass application.

**Figure 3:**
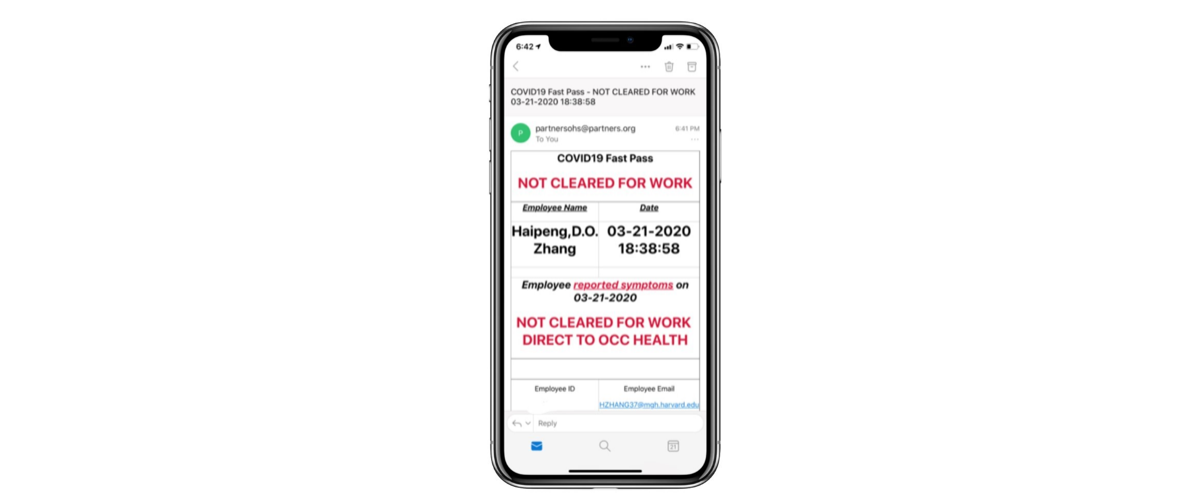
“Not Cleared for Work” screen of the COVID Pass application.

**Figure 4:**
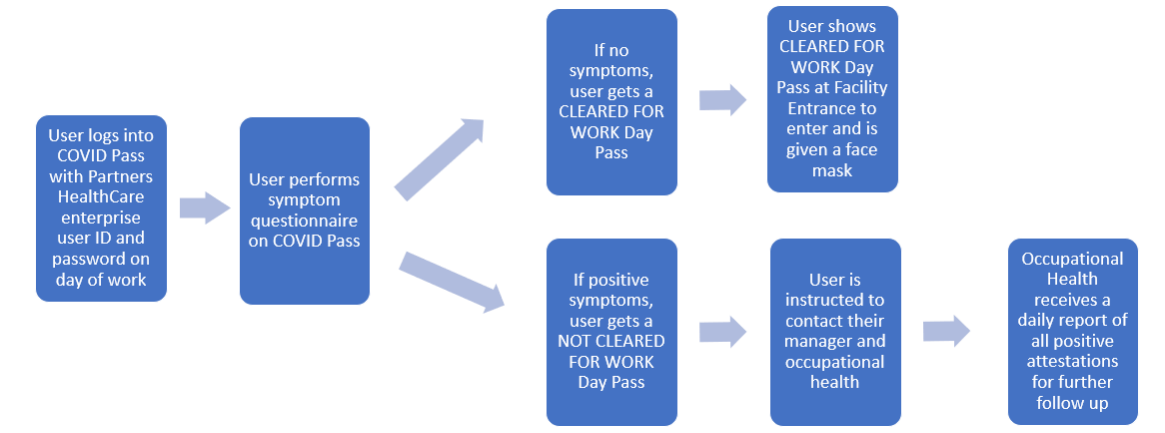
Schematic of the process and procedures involved in using the COVID Pass application.

The self-service application, initially developed in REDCap, was called COVID Pass. The application required users to log in with a Partners HealthCare network user ID and password. The application then authenticated the user against Active Directory and used the login ID to look up the employee’s first name, last name, email address, and employee ID. The employee would review this information and select whether they had symptoms of COVID-19 from a list of symptoms determined by infection control leaders (Figure 1). Some symptoms were marked as NEW in the system to better differentiate chronic symptoms from acute onset symptoms. If the employee selected “no symptoms,” they were required to attest to this with their initials and were then provided a pass to enter the facility for the day. The pass was displayed on the screen, and a copy was automatically sent to the employee’s email address (Figure 2). Employees indicating one or more symptoms were directed not to come to work and to call their manager and Occupational Health Services (Figure 3). The occupational health team would receive a daily report of positive symptom attestations and follow up according to their standard protocols. A copy of the relevant information was also sent to the employee’s email address. Figure 4 shows a schematic of the processes and procedures involved in using the self-service component of COVID Pass.

We made COVID Pass available to employees via multiple methods. Working with marketing and

internal communications experts, we selected an easy-to-remember URL,[9]. We also created a quick response (QR) code for the URL that that users could scan with their smartphones to be directed to the website. Finally, we wrapped the website in native iOS and Android apps so that the application could be distributed internally through our employee App Catalog.

For employees who did not have access to a computer or mobile device to complete COVID Pass, a manual process was established where in-person, socially distanced screeners asked employees entering the site if they had symptoms and then manually recorded the responses. For the manual pathway MVP, we created custom Microsoft Office 365 forms (Microsoft Corporation) that enabled screeners at each of the pilot sites to quickly record the employee’s first and last name as well as whether they had symptoms.

**Figure 5:**
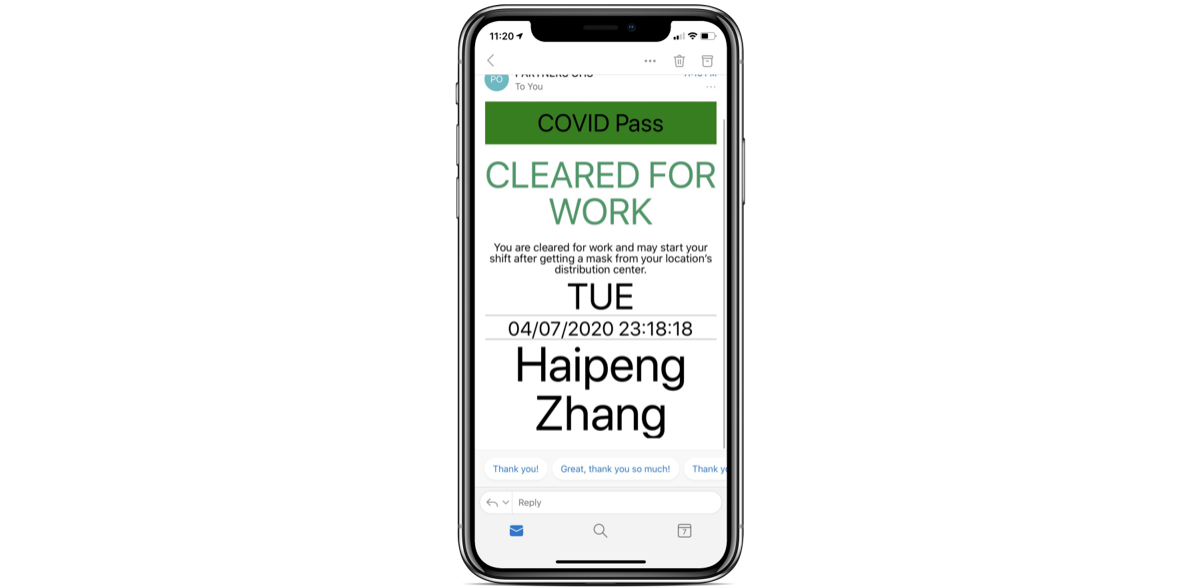
Screenshot of the .NET Framework version of COVID Pass.

A custom .NET Framework application (Microsoft Corporation) was developed by a separate development team as the REDCap version of COVID Pass was being refined and its requirements solidified (Figure 5). In this version, both the self-service attestation mode and the manual process were rolled into one build. In addition, a kiosk mode version of COVID Pass was created for employees to provide attestations at the entrances of facilities. As COVID Pass scaled, we ultimately transitioned from the REDCap application to the custom .NET application on March 30, 2020, at 5 PM.

All versions of COVID Pass recorded employee attestations and stored them in one central database. The data were aggregated at the site level and shared daily with site operational leaders; names of individuals reporting positive symptoms were shared with Human Resources leadership daily. In this report, we share three months of data from COVID Pass use from March 30, 2020, to June 30, 2020.

Aggregate descriptive statistics, including overall employee attestations by day and site, employee attestations by application method (COVID Pass automatic screening vs manual screening vs kiosk), employee attestations by time of day, and percentage of employees reporting COVID-19 symptoms, were compiled using SAS (Enterprise Guide 7.1, SAS Institute Inc) and Tableau (Desktop 2020.2, Tableau Software LLC). This study was reviewed by the Partners HealthCare Institutional Review Board and deemed to not meet the definition of human subjects research.

**Figure 6:**
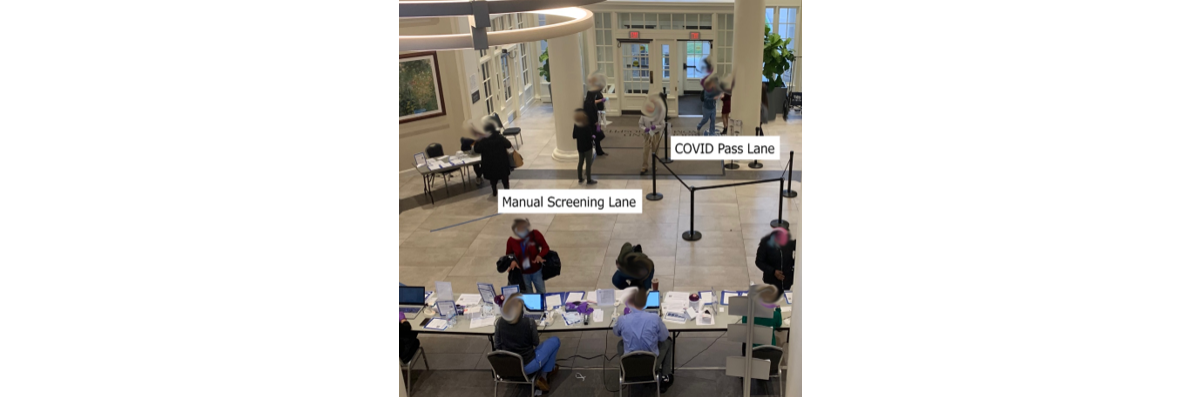
Hospital staff entrance showing the COVID Pass lane for employees who used the COVID Pass application and the manual screening lane for employees who did not use the application.

The COVID Pass MVP was initially tested at two hospitals within our system on March 23, 2020. As part of this controlled rollout, the project team participated in on-site testing of the application, gathering user feedback and being “at the elbow” with the staff at the entranceways of the sites. Figure 6 shows an image of a hospital entrance with a COVID Pass lane for employees who completed COVID Pass prior to or immediately upon arrival. The manual screening lane includes a table at which screeners can enter the employees’ attestations manually. Feedback from both staff assigned to screen employees and employees using COVID Pass was relayed to the developer for further refinement of the application. In parallel to this development process, the number of sites within the system using COVID Pass was slowly increased throughout the week. By March 30, 2020, COVID Pass was deployed across the enterprise.

Documentation about the initiation of COVID Pass within a facility was collated and distributed to the sites as new COVID Pass sites went live. This documentation included standard language, collateral such as flyers used in previous site implementations, and “lessons learned” from earlier implementations. The collective documentation also simplified the process of scaling this solution across the enterprise.

## Results

**Figure 7:**
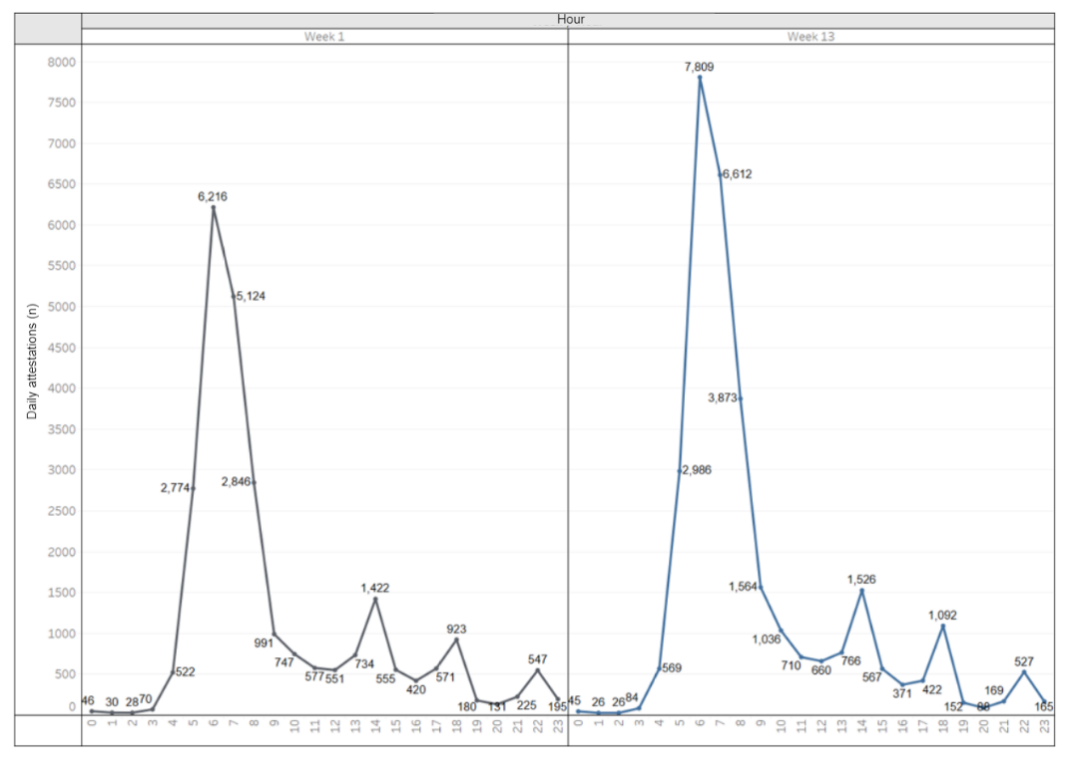
Average daily employee attestations from Monday to Friday (COVID Pass, manual screening, and kiosk) by hour of day during Week 1 (March 30 to April 3, 2020) and Week 13 (June 22 to 26, 2020).

Multimedia Appendix 1 summarizes the number of employee attestations (using either COVID Pass, the manual process, or kiosks) by week and site. 1,976,379 of the 2,169,387 total attestations (91.1%) were generated from the self-service screening application. The remainder (Table 1) were generated either from manual attestation processes (174,865/2,169,387, 8.1%) or COVID Pass kiosks (25,133/2,169,387, 1.2%). Hospital staff continued to work 24 hours per day, with peak staff attestations during shift changes between 6 and 8 AM, 1 and 3 PM, 5 and 7 PM, and 10 PM and midnight (Figure 7).

As the number of COVID-19 cases stabilized in the region, more employees transitioned back to working on-site. Figure 7 presents a comparison between the average daily (Monday to Friday) number of hourly attestations in week 1 (week of March 30) vs week 13 (week of June 22). By week 13, the average number of attestations at the peak 6 AM shift change had increased by 1593 (25.63%).

There was an overall increase of 26,179 attestations weekly by week 13 compared to week 1, representing a 14.46% increase in attestations.

**Table 1:** COVID Pass attestations by employee app-based self-attestation, manual screening, and kiosk by site on March 30 to June 30, 2020 (N=2,169,387). Note that some sites used separate manual screening data collection methods; data for these sites are not reflected here.

Site number (total attestations, n)	Attestation source, n (%)		
	App	Kiosk	Manual
1 (732,278)	713,259 (97.4)	1552 (0.2)	17,467 (2.4)
2 (564,710)	497,101 (88.0)	16,129 (2.9)	51,480 (9.1)
3 (166,985)	140,964 (88.4)	2466 (1.5)	23,555 (14.1)
4 (148,918)	127,010 (85.3)	133 (0.1)	21,775 (14.6)
5 (107,191)	104,368 (97.4)	0 (0.0)	2823 (2.6)
6 (93,142)	70,124 (75.3)	4763 (5.1)	18,255 (10.6)
7 (66,581)	64,838 (97.4)	18 (<0.1)	1725 (2.6)
8 (57,773)	51,260 (88.7)	0 (0.0)	6513 (11.3)
9 (50,552)	34,845 (68.9)	0 (0.0)	15,707 (37.9)
10 (47,532)	44,650 (93.9)	4 (<0.1)	2878 (6.1)
11 (32,766)	32,080 (97.9)	22 (0.1)	664 (2.0)
12 (32,176)	24,010 (74.6)	0 (0.0)	8166 (23.4)
13 (25,744)	24,934 (96.9)	0 (0.0)	810 (3.1)
14 (15,713)	13,438 (85.5)	1 (<0.1)	2274 (14.5)
15 (14,132)	13,654 (96.6)	0 (0.0)	478 (3.4)
16 (8437)	8400 (99.6)	1 (<0.1)	36 (0.4)
17 (1837)	1803 (98.1)	31 (1.7)	3 (0.2)
18 (1048)	896 (85.5)	0 (0.0)	152 (14.5)
19 (658)	568 (86.3)	6 (0.9)	84 (12.8)
20 (643)	643 (100.0)	0 (0.0)	0 (0.0)
21 (571)	534 (93.5)	7 (1.2)	30 (5.3)
All sites (2,169,387)	1,969,379 (90.8)	25,133 (1.2)	174,875 (8.1)

## Discussion

### Principal Findings

Within two weeks of conception, COVID Pass went live across the entire Partners HealthCare enterprise. During weekdays, COVID Pass recorded over 25,000 attestations per day. This high rate of adoption would not have occurred at such a fast pace without the following key considerations: mandatory and incentivized use while minimizing friction; planning for accessibility; leveraging REDCap to enable an agile development process; rapid analysis and distribution of COVID Pass data; and pre-emptive transition to a more durable and scalable platform.

By making COVID Pass mandatory for employees to gain access to their work sites and incentivizing it by pairing it with the distribution of masks, we quickly increased the acceptability of the potential burden of mandatory prework self-attestation. We believed that COVID Pass would further benefit from minimization of friction during completion of the attestation process. We achieved this by minimizing the number of interactions users needed to perform to complete the COVID Pass process while maximizing the data captured. By autopopulating demographic information after login, we only required the user to answer one question (symptom review). If the user was asymptomatic, we required them to attest to this with their initials to receive their COVID Pass for the day. We also simplified access to COVID Pass through the creation of multiple distribution channels, including a simple URL that was sent with all communication to staff throughout our organization, a QR code that was used on flyers in the entranceways of our facilities, and iOS and Android app versions of COVID Pass that were made available through our employee-facing App Catalog.

As part of this work, we also knew that we would need to provide an accessible pathway for users who might not be able to complete the self-service COVID Pass due to limited proficiency with a smartphone or computer, language limitations, or other reasons. By incorporating this accessibility requirement early in our development process and creating a manual pathway, we ensured that COVID Pass would be a comprehensive solution for all employees. Eventually, we also added a kiosk mode for COVID Pass, along with multilingual support to further enhance accessibility.

We adopted an agile development process to further minimize friction for the end user through early on-site testing and rapid iteration cycles. An agile development approach emphasizes “early and continuous delivery of valuable software” while accommodating changing requirements as the software is used in the real world and assumptions are validated or invalidated [10]. Our team created the MVP for COVID Pass within 48 hours and began testing the solution the very next day. We refined the requirements for COVID Pass and made updates to the MVP multiple times throughout the day at the beginning of this process.

Much of this early ability to create rapid changes and adjustments to COVID Pass came from the initial platform decision to use REDCap for the MVP. As the core functionality of REDCap was able to accommodate most of the initial requirements of COVID Pass, the symptom survey fields and conditional text could be adjusted almost instantaneously. This enabled our team to sustain a rapid iteration cycle during the first week of deployment while additional sites across the organization went live with COVID Pass.

In addition, REDCap enabled our team to rapidly export data and custom reports from COVID Pass to our organization daily. These data were primarily used to perform two major functions within the organization: (1) tracking symptomatic employees for further workup of COVID-19 by occupational health and (2) estimating the number of employees on site for personal protective equipment (PPE) supply planning.

Within the first week, COVID Pass had identified over 500 employees with symptoms suggestive of COVID-19 infection. After three months, COVID Pass had identified 1865 employees with symptoms. These employees were all instructed to contact Occupational Health Services for next steps. In addition, all employees with positive symptoms were flagged to be contacted by Occupational Health Services for further questioning and triage. Further work is needed to determine how many of these employees were actually confirmed to have COVID-19.

The reproduction number of COVID-19 has been preliminarily estimated to be between 2 and 4 [11,12]. By identifying 1865 employees with symptoms suggestive of COVID-19 and instructing them to not report to work, and by alerting Occupational Health Services about these employees for further follow-up, COVID Pass likely played an important role in limiting further spread of COVID-19 within our workforce, our patients, and the larger community in our region.

The World Health Organization estimates that up to 89 million medical masks are required monthly for the COVID-19 response [13]. As all COVID Pass users who are cleared for work are issued a face mask, the daily logs from COVID Pass have become an important proxy for the number of masks being distributed daily to employees within our organization. This also provides leaders within the organization with a good approach to predict face mask allocations per facility per day and plan accordingly.

By the middle of the first week of the COVID Pass rollout, it became apparent that a more hardened version of this application would be needed. Some of the clear advantages of REDCap, such as the prebuilt scaffolding to support survey creation and data export, became limitations as more custom change requests began to surface for COVID Pass. In addition, as COVID Pass was rapidly adopted across the enterprise, a more formalized support structure was needed to maintain the operation of COVID Pass 24 hours per day, 7 days per week. As a result, we worked in parallel with a second, in-house development team to create a custom version of COVID Pass built on the Microsoft .NET Framework with a structured query language (SQL) database, using the lessons learned and updated requirements gained through the release of the live REDCap application.

We ultimately transitioned to the .NET version of COVID Pass on March 30, 2020, at 5 PM. The process of developing the same solution in parallel and on two different platforms allowed us to leverage the strengths of each one and achieve a more robust final product. The established processes and large user base for COVID Pass meant that a compiled, single-purpose application, such as the .NET application, was better suited for the long-term needs of the project. Transitioning the application to an infrastructure environment that provided 24/7 support and available resource capacity enabled the system to handle high load times during the morning hours, when the highest number of concurrent users occurs. A refined self-service module and a manual pathway built on the same platform enabled streamlining of the data collection and analysis as well.

### Limitations

We custom-developed COVID Pass to meet local requirements for employee symptom attestation. Other organizations may have different operational requirements. We are making both the REDCap and .NET source code available to other organizations to use and modify as needed. Secondly, some of our sites did not track manual attestation using the Office 365 forms or .NET applications; therefore, we do not possess complete data on manual screenings. Finally, as the scope of this paper is focused on the data generated directly from the COVID Pass applications, we have no data on the number of positive or negative COVID-19 tests given to employees who were flagged by COVID Pass to Occupational Health Services.

### Conclusion

Health systems worldwide have faced incredible challenges due to the COVID-19 pandemic. At Partners HealthCare, one challenge was the evolving role of symptom monitoring for all employees working on-site. Technology is not a panacea; however, when it is used appropriately and scoped to the right problems, technology can play a meaningful role in the era of COVID-19.

With the combination of a rapid, agile approach to software development and a use case paired with an end-user incentive, we quickly created and deployed a dedicated employee attestation application that gained widespread adoption and use within our health system. COVID Pass is continuing to support daily screening for over 30,000 employees. Further, we identified 1865 symptomatic employees who otherwise may have come to work, potentially putting others at risk.

We continue to use the data obtained through COVID Pass for incident planning, such as PPE supply. The COVID Pass platform also has potential to be an effective communication tool to the workforce; one use case that we are exploring is using the final page of COVID Pass to inform employees about voluntary COVID-19 research studies within our institution.

As we share these lessons learned and the story of our implementation of COVID Pass, we also want to ensure that other institutions that may want to implement a similar solution can not only learn from our implementation but can also access the source code for COVID Pass. Therefore, we are making the source code for COVID Pass available to all via GitHub [14].

We are living through extraordinary times. We are confident that the bravery, commitment, and perseverance of the clinicians and hospital staff on the front lines of this worldwide crisis will help see us safely through the COVID-19 pandemic. Our hope is that software solutions such as COVID Pass will play their own small roles within this larger effort and that others may make meaningful use of this tool, as our institution has.

## Appendix

Multimedia Appendix 1 Weekly completed employee symptom attestations (COVID Pass and manual) by day and site.

## Abbreviations

MDPH: Massachusetts Department of Public Health
MVP: minimum viable product
PPE: personal protective equipment
QR: quick response
REDCap: research electronic data capture
SQL: structured query language
