# Supplementary material for: A Web-Based, Mobile-Responsive Application to Screen Health Care Workers for COVID-19 Symptoms: Rapid Design, Deployment, and Usage
Source: JMIR Form Res. 2020 Oct 8;4(10):e19533. doi: 10.2196/19533 (PMC7546861; doi:10.2196/19533)
Supplement: Multimedia Appendix 1 [file formative_v4i10e19533_app1.docx]

**Supplementary Table. Weekly completed employee symptom attestations (COVID Pass and manual) by day and site.**

| Site number | Week 1 | Week 2 | Week 3 | Week 4 | Week 5 | Week 6 | Week 7 | Week 8 | Week 9 | Week 10 | Week 11 | Week 12 | Week 13 | Week 14 | Total |
| --- | --- | --- | --- | --- | --- | --- | --- | --- | --- | --- | --- | --- | --- | --- | --- |
| 1 | 49,242 | 50,091 | 51,079 | 51,511 | 50,659 | 49,813 | 50,816 | 51,800 | 49,909 | 60,473 | 62,832 | 64,644 | 65,649 | 23,760 | 732,278 |
| 2 | 41,019 | 39,878 | 39,789 | 40,014 | 39,745 | 39,318 | 39,992 | 40,964 | 38,589 | 45,529 | 46,934 | 47,953 | 47,739 | 17,247 | 564,710 |
| 3 | 12,866 | 12,898 | 12,945 | 12,533 | 12,634 | 12,354 | 12,444 | 12,406 | 11,459 | 12,447 | 12,783 | 12,362 | 12,473 | 4,381 | 166,985 |
| 4 | 12,050 | 11,067 | 11,089 | 10,514 | 10,776 | 10,628 | 10,768 | 11,025 | 10,385 | 11,490 | 11,530 | 11,762 | 11,549 | 4,285 | 148,918 |
| 5 | 7691 | 7568 | 7462 | 7257 | 7134 | 7504 | 8148 | 8421 | 7561 | 8809 | 8843 | 8746 | 8735 | 3312 | 107,191 |
| 6 | 6751 | 6764 | 6903 | 7011 | 6819 | 6574 | 6721 | 6775 | 6422 | 7454 | 7376 | 7484 | 7491 | 2597 | 93,142 |
| 7 | 4861 | 4933 | 4945 | 5031 | 4963 | 4964 | 4936 | 4898 | 4477 | 5149 | 5223 | 5223 | 5145 | 1833 | 66,581 |
| 8 | 4452 | 4538 | 4421 | 4105 | 4360 | 4224 | 4194 | 4084 | 3881 | 4392 | 4448 | 4471 | 4606 | 1597 | 57,773 |
| 9 | 3756 | 3663 | 3625 | 3654 | 3711 | 3724 | 3606 | 3600 | 3552 | 3917 | 3985 | 4059 | 4227 | 1473 | 50,552 |
| 10 | 3338 | 3491 | 3474 | 3425 | 3484 | 3479 | 3632 | 3714 | 3400 | 4139 | 3582 | 3424 | 3523 | 1427 | 47,532 |
| 11 | 2012 | 2671 | 2612 | 2552 | 2545 | 2473 | 2464 | 2480 | 2122 | 2461 | 2441 | 2507 | 2502 | 924 | 32,766 |
| 12 | 1715 | 2315 | 2403 | 2450 | 2438 | 2411 | 2533 | 2543 | 2324 | 2548 | 2538 | 2635 | 2511 | 812 | 32,176 |
| 13 | 2129 | 1977 | 2079 | 2050 | 1924 | 1897 | 1863 | 1833 | 1557 | 1824 | 1896 | 1976 | 1954 | 785 | 25,744 |
| 14 | 1064 | 1170 | 1165 | 1189 | 1104 | 1156 | 1216 | 1183 | 1117 | 1228 | 1227 | 1250 | 1239 | 405 | 15,713 |
| 15 | 956 | 1027 | 995 | 991 | 997 | 1008 | 1020 | 1069 | 987 | 1134 | 1120 | 1181 | 1201 | 446 | 14,132 |
| 16 | 0 | 38 | 361 | 1524 | 1475 | 1472 | 1304 | 1045 | 735 | 260 | 87 | 62 | 51 | 23 | 8437 |
| 17 | 0 | 0 | 0 | 124 | 246 | 241 | 249 | 235 | 181 | 127 | 124 | 130 | 142 | 38 | 1837 |
| 18 | 76 | 72 | 60 | 71 | 71 | 48 | 64 | 54 | 55 | 126 | 98 | 103 | 101 | 49 | 1048 |
| 19 | 59 | 83 | 65 | 41 | 47 | 78 | 40 | 39 | 25 | 32 | 42 | 41 | 45 | 21 | 658 |
| 20 | 643 | 0 | 0 | 0 | 0 | 0 | 0 | 0 | 0 | 0 | 0 | 0 | 0 | 0 | 643 |
| 21 | 54 | 35 | 28 | 46 | 42 | 49 | 48 | 40 | 34 | 42 | 41 | 43 | 49 | 20 | 571 |
| Total | 154,734 | 154,279 | 155,500 | 156,093 | 155,174 | 153,415 | 156,058 | 158,208 | 148,772 | 173,581 | 177,150 | 180,056 | 180,932 | 65,435 | 2,169,387 |
